# Bilateral double beta peaks in a PD patient with STN electrodes

**DOI:** 10.1007/s00701-020-04493-5

**Published:** 2020-07-24

**Authors:** Thomas Koeglsperger, Jan H. Mehrkens, Kai Bötzel

**Affiliations:** 1 Department of Neurology, Ludwig Maximilian University, Marchioninistr, 15, 81377 Munich, Germany; 2 Department of Neurosurgery, Ludwig Maximilian University, Marchioninistr, 15, 81377 Munich, Germany

**Keywords:** Deep brain stimulation (DBS), Local field potential (LFP), Beta band frequency, Closed loop, Kinematic sensor

## Abstract

Subthalamic local field potentials in the beta band are considered as potential biomarkers for closed-loop deep brain stimulation. To investigate the subthalamic beta band peak amplitudes in a Parkinson’s disease patient over an extended period of time by using a novel and commercially available neurostimulator with permanent sensing capability. We recorded local field potentials of the subthalamic nucleus using the Medtronic Percept™ implantable neurostimulator at rest and during physical activity (gait) with and in response to deep brain stimulation. We found a double-peaked beta activity on both sides. Increasing stimulation and physical activity resulted in a decreased beta band amplitude, but was accompanied by the appearance of a second, and previously unrecognized peak at 13 Hz in the right hemisphere. Our results will support the investigation of distinct different peaks in the beta band and their relevance and usefulness as closed-loop biomarkers.

**Electronic supplementary material:**

The online version of this article (10.1007/s00701-020-04493-5) contains supplementary material, which is available to authorized users.

## Introduction

Subthalamic nucleus (STN) local field potentials (LFPs) represent an electrophysiological correlate of a patient’s clinical symptoms in Parkinson’s disease (PD) [5]. Previous research has particularly investigated the beta band (13–30 Hz) of LFPs, since peaks in this frequency spectrum correlate with bradykinesia and respond to treatment with l-Dopa and deep brain stimulation (DBS) [2, 4, 5]. Previous DBS devices did not allow for long-term recording, making it difficult to measure LFPs over an extended period of time. Here, we recorded continuous LFPs from the implantable pulse generator (IPG; Medtronic Percept™) in a PD patient and found that this particular patient exhibited two distinct peaks in the beta-band LFP power on either side. These peaks were recorded during intraoperative recordings as well as chronically with the implantable device and were suppressed by stimulation and gait, whereas a third peak on the right side emerged only in response to stimulation.

## Material and methods

The present data were obtained from a 56-year-old man who had suffered from PD of the equivalence type for the past 6 years. One year prior to surgery, he developed severe on-off fluctuations. He never experienced falls, gait-freezing, or signs of cognitive decline. In order to ameliorate his symptoms, he underwent DBS surgery in January 2020. Electrode leads (Medtronic 3389™) were implanted into the dorsolateral aspect of the STN on both sides. Single cell activity and LFPs were recorded during the surgery and test stimulation was applied to evaluate its effect on rigidity and bradykinesia. The pulse generator was implanted on the 3rd postoperative day (Medtronic Percept™) and stimulation initiated and optimized in the following 8 weeks. Within the scope of the present work, LFP traces were recorded from the electrodes adjacent to the respective stimulation electrode on each side during rest and gait (left STN: stimulation at contact #1, sensing at contacts #0 and #2; right STN: stimulation at contact #10, sensing at contacts #9 and #11). Gait performance was continuously monitored with sensors attached to the shanks, thighs, arms, and chest, and step length, cadence, and foot clearing were measured as described [1]. Gait recordings and LFPs were synchronized by detecting the impulses of a transcutaneous electric nerve stimulator triggered by the gait recording devices. The exact timing and amplitude of stimulation was recorded by the stimulator and transferred to the tablet computer. All tests were approved by the local ethics committee (Project No.: 17-639) and were in accordance with the declaration of Helsinki. UPDRS III scores were obtained preoperatively and postoperatively. During the tests, the raw LPSs (one channel from each electrode lead) were gained up by 250× and streamed wirelessly to a tablet computer. The sampling rate was 250 Hz. Further details of the recording technique are described elsewhere [8]. The stimulator computes the spectrum of the LFPs every 500 ms and evaluates peak amplitudes in a user-defined frequency band (here: 20–30 Hz). This peak amplitude evaluation is performed by an on-chip digital fast Fourier transform circuit. Amplitude information is reported in user-defined center frequency ± 2.5 Hz. These data were also real time streamed wirelessly to the tablet computer. Further evaluation was done with Matlab: LFP spectra were computed for 512 data points with an overlap of 256 points and averaged to obtain spectra for epochs of 5 s. Peak amplitudes for each of these 5 s-segments were measured as the maximum of the spectrum in the range of 12–20 Hz (low beta band) and 20–30 Hz (high beta band).

## Results

**Fig. 1:**
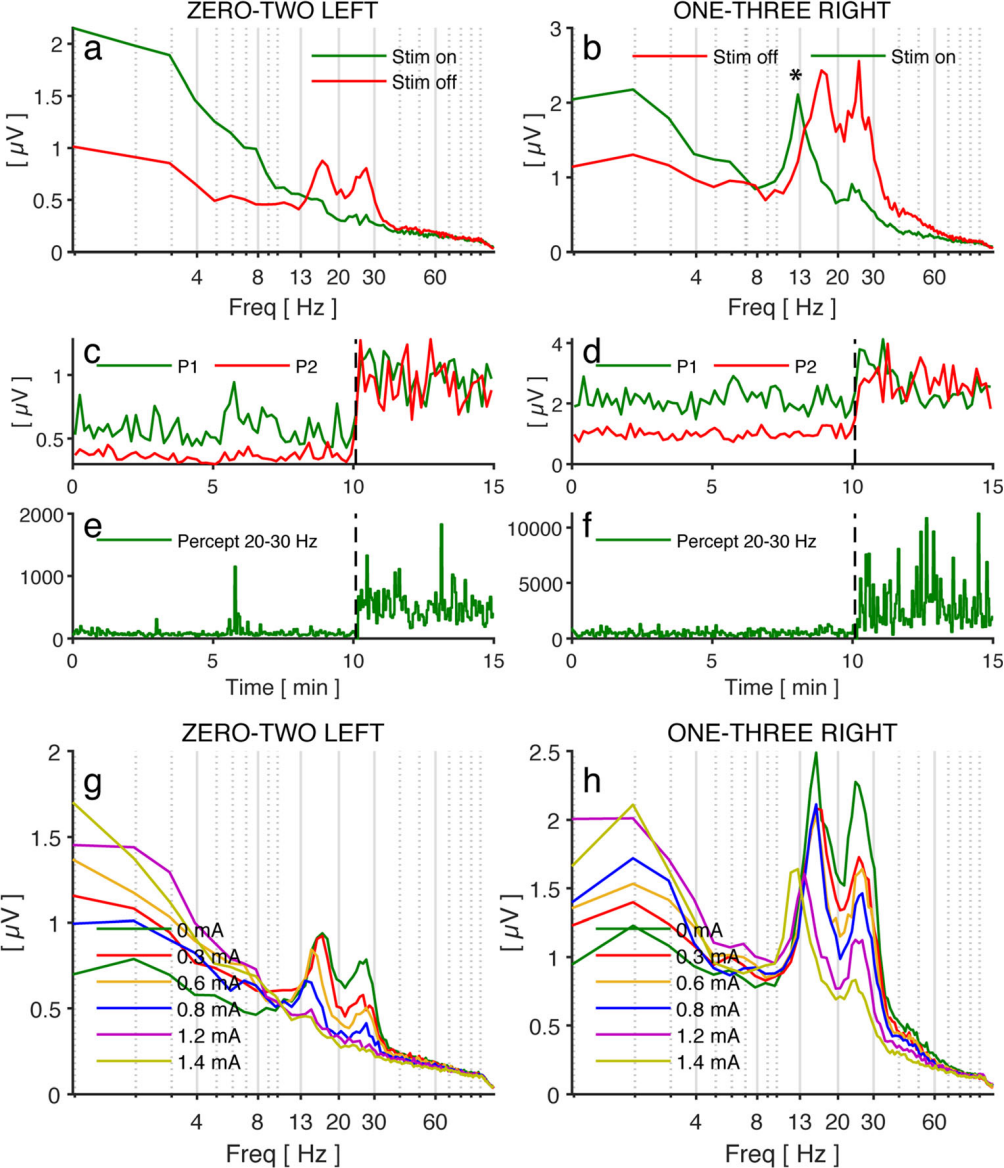
**a**, **b** Frequency spectrogram at the beginning (at 0 min, green, stim-off) and the end (at 15 min, red, stim-on) of the recording. Note the appearance of a low-frequency 13-Hz peak in response to stimulation on the right electrode (asterisk). **c**, **d** Peak amplitude at 15 Hz (P1, green) and 25 Hz (P2, red) over time computed off-line by Matlab. Stimulation has been stopped at *t* = 10 min (dashed vertical line). Note that the beta amplitude on the right side (P1, green line) shows no lasting decrement in response to stimulation onset due to the presence of a novel 13-Hz peak. **e**, **f** Peak amplitude measured between 20 and 30 Hz over time as computed in real time by the neurostimulator. Because of the high-frequency spectrum, the appearance of the low beta peak at 13 Hz has no effect. **g**, **h** Frequency spectra during stim-off and in response to gradual increase of the stimulation amplitude until a clinically efficient stimulation threshold was reached (1.4 mA, 130 Hz, 60 μs). Recordings from the left electrode showed a gradual peak amplitude reduction with increasing stimulation. On the right side, only the peak at 25 Hz decreased in response to stimulation, whereas the peak at 15 Hz was replaced by a peak at 13 Hz when stimulation was increased from 0.8 to 1.2 mA. Stimulation was increased every 2 min (0 mA, 0.3 mA, 0.6 mA, 0.8 mA, 1.2 mA, 1.4 mA). Electrode configuration is indicated above each image: zero-two left = stim at contact #1, sensing from contact #0 and 2, left hemisphere; one three right = stim at contact #2, sensing from contact #1 and 3, right hemisphere

**Fig. 2:**
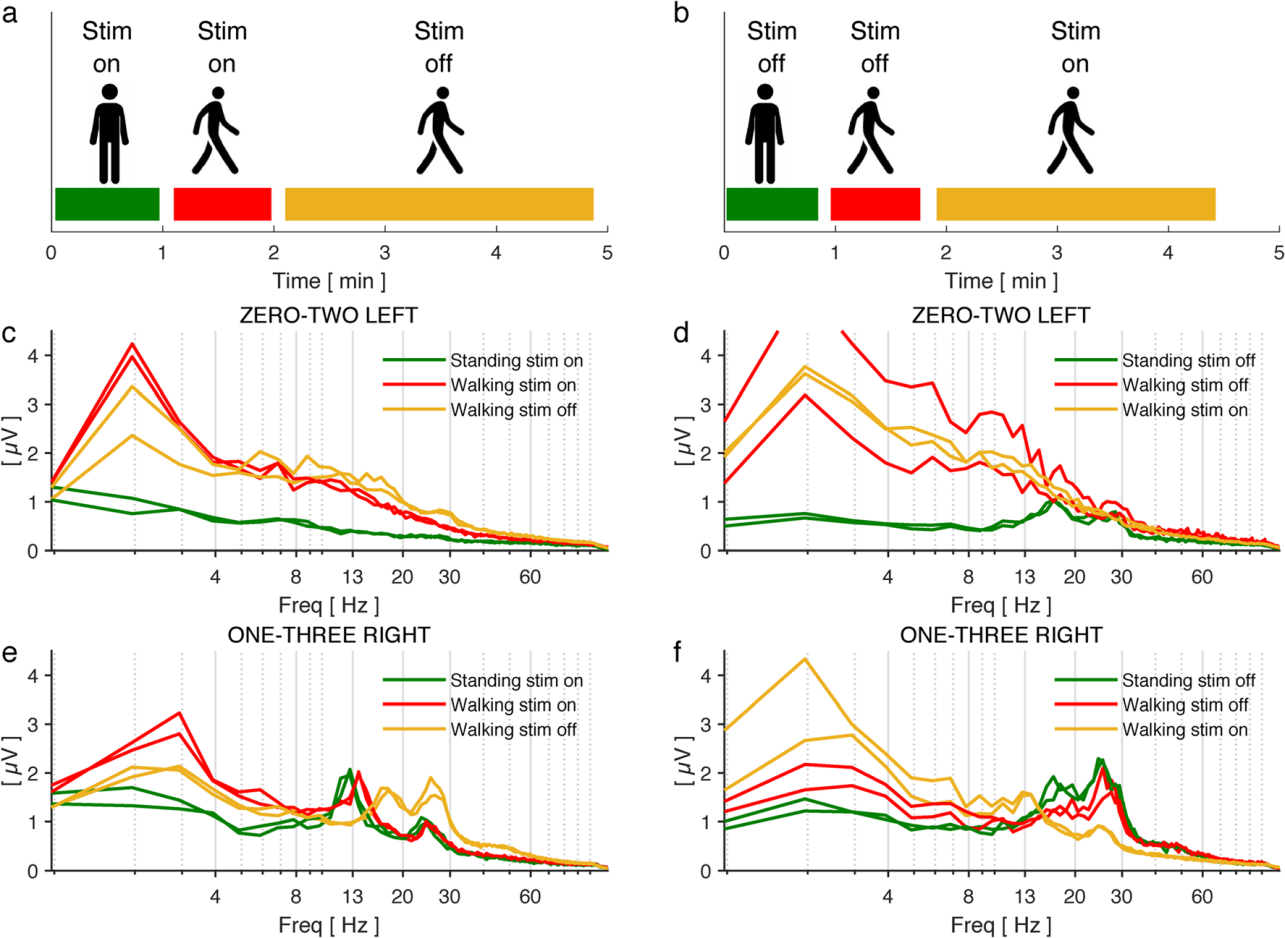
**a**, **b** Schematic illustrating the test sequence. The patient was subsequently asked to stand for 1 min and then walk for 300 m. Stimulation was turned off after 1-min walking. Next, the patient was asked to stand for another 1 min and then walk again for 300 m, during which the stimulation was switched on wirelessly after 1 min. **c**–**f** Frequency spectrograms obtained during the respective gait and stimulation period. Each part of the test was reproduced once and the results represented as duplicates. The colors of the different curves correspond to the respective periods in **a** and **b**. Note the differences between the spectra of the left (**c**, **d**) and right (**e**, **f**) electrode. Electrode configuration is indicated above each image: zero-two left = stim at contact #1, sensing from contact #0 and 2, left hemisphere; one three right = stim at contact #2, sensing from contact #1 and 3, right hemisphere

LFPs recorded intraoperatively showed one peak amplitude in the beta band on the left side and two distinct peak amplitudes on the right side (Supp. Fig. 1a, b). Once the IPG was implanted and its settings were optimized, stimulation led to a remarkable improvement of the patient’s symptoms (UPDRS III stim off: 38, med-on: 16, stim-on and med-off: 7). The spectra of the LFP showed a double-peaked beta activity on both sides (at 15 and 25 Hz) (Fig. 1a, b; red trace), which was suppressed by stimulation on the left side, whereas on the right side, a novel peak appeared in the low beta band in response to stimulation (asterisk) (Fig. 1a, b; green trace). The DBS effect was readily appreciated when the individual peaks (15 Hz and 25 Hz) were analyzed separately over time (Fig. 1c, d), but was not found in a compound analysis done by the stimulator that computes the beta peak amplitude between 20 and 30 Hz (Fig. 1e, f). When we analyzed the LFP peaks in response to different stimulation amplitudes, we found a gradual reduction of both beta peaks on the left side with increasing stimulation amplitudes (Fig. 1g). Conversely, on the right side, this was only true for the peak at 25 Hz, whereas the low beta peak at 15 Hz was replaced by a peak at 13 Hz when stimulation was increased from 0.6 to 0.8 mA (Fig. 1h). In addition to these experiments, we tested the effect of gait on LFPs in the same patient (Fig. 2). The LFP spectra recorded during gait were evaluated in three distinct intervals: standing (with/without stim), walking (stim on), and walking (stim off) (Fig. 2a, b). On the left side, stimulation caused a complete suppression of beta peaks; gait suppressed beta peaks almost completely when stimulation was off (Fig. 2c, d). However, on the right side, stimulation caused only a partial suppression of beta peaks as well as the appearance of an additional peak at 13 Hz. Gait had an additional but incomplete suppressive effect on beta peaks on the right side during gait under stimulation (Fig. 2e, f).

## Discussion

This single case study illustrates the prospects of using novel neurostimulation devices that allow continuous recording of LFPs in addition to delivering DBS to the respective target structure. As compared with previous studies that recorded beta peaks in PD patients temporally, our results suggest a more complex behavior of these beta peaks in response to DBS and to gait. In this regard, several aspects merit particular consideration: first, two beta peaks were seen on either side and behaved similarly with respect to stimulation and movement on the left side, i.e., showed an almost complete disappearance. However, on the right side, only the high beta peak reacted with a moderate amplitude reduction to both gait and stimulation, whereas the low beta peak disappeared with higher stimulation and was replaced by a 13-Hz peak, which has not been described previously. Numerous previous studies have not described low and high beta peaks, thus possibly neglecting the presence of two distinct peaks in this part of the spectrum. In the literature, there is still limited evidence for a different behavior of low and high beta peaks: low beta peaks were associated with freezing of gait [7, 9] and react differently to dopaminergic treatment [6]. In our study, movement (gait) suppressed beta peaks on the left side almost completely but caused only a partial amplitude decrease of beta activity on the right side. These observations have important implications for LFP-controlled closed-loop stimulation and future studies need to address the specific beta peaks and their behavior in response to DBS. In a recent paper, beta characteristics of motor subtypes (tremor dominant, gait-related) were described [10]. The identification of these unique electrophysiological “fingerprints” and their connection to motor subtypes will likely support the investigation of LFPs for closed-loop stimulation. For instance, when movement causes a suppression of beta peaks as seen in our recordings of the left lead, this may erroneously be interpreted as absence of bradykinesia by the algorithm [3]. When stimulation results in an additional peak, as seen in our recordings (Fig. 2) of the right lead, this may likewise affect the usefulness of LFPs as a feedback signal for closed-loop stimulation.

## Conclusions

In summary, our results will support the investigation of patient-specific individual LFP patterns (LFP “fingerprints”) and aid the development of LFP-based feedback signals. In addition, our exploratory study highlights the potential of dual deep brain stimulation and recording devices (DBS/R) for the study of LFPs.

## Electronic supplementary material

Supplementary Fig. 1 LFP spectra obtained at different depths of the left and right STN are shown. Depth (mm) indicates the electrode tip (1.5 mm below ring electrode used for LFP recordings). On the left, a broad beta peak can be seen. On the right side, two beta beaks were recorded over the whole recording distance. (PNG 2961 kb)

High resolution image (TIFF 3876 kb)
