# Supplementary figures and images for: Bilateral double beta peaks in a PD patient with STN electrodes
Source: Acta Neurochir (Wien). 2020 Jul 24;163(1):205–9. doi: 10.1007/s00701-020-04493-5 (PMC7778623; doi:10.1007/s00701-020-04493-5)

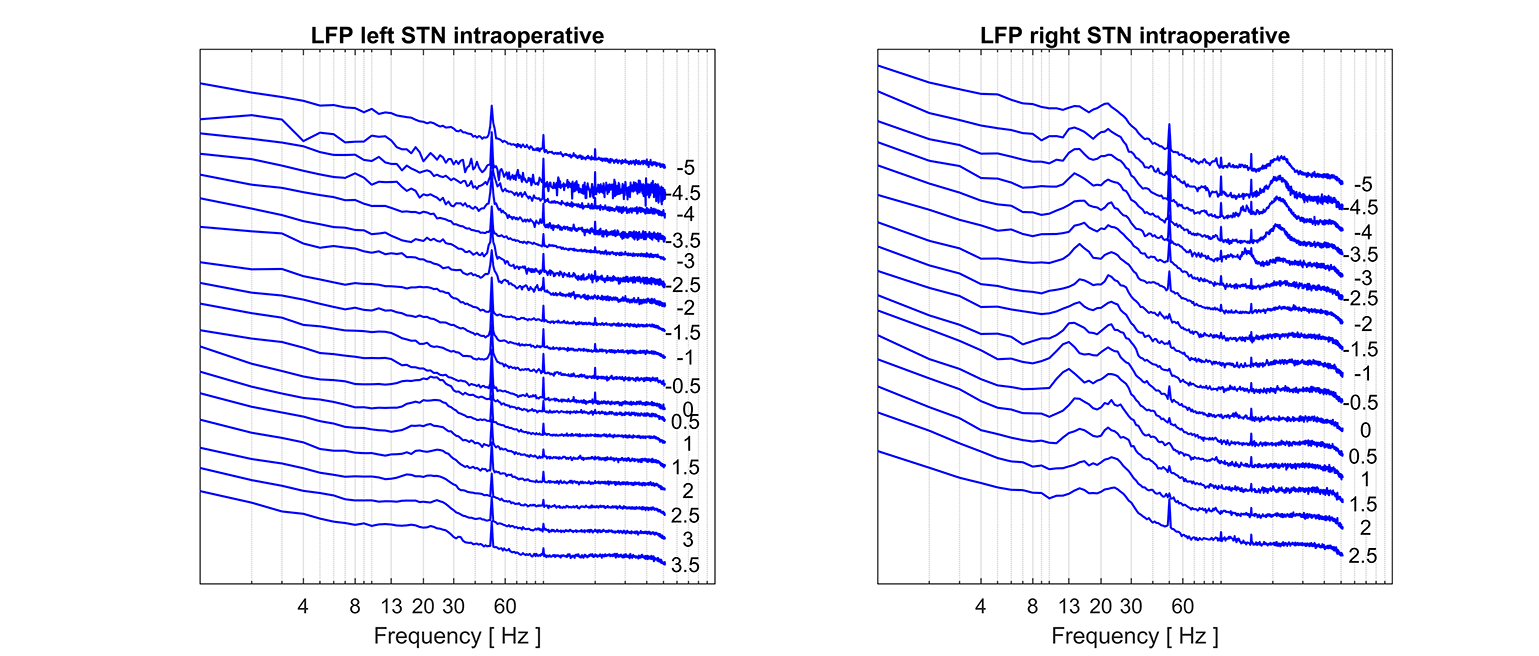

Supplement: Supplementary file 1 — LFP spectra obtained at different depths of the left and right STN are shown. Depth (mm) indicates the electrode tip (1.5 mm below ring electrode used for LFP recordings). On the left, a broad beta peak can be seen. On the right side, two beta beaks were recorded over the whole recording distance. (PNG 2961 kb) [file 701_2020_4493_Fig3_ESM.png]

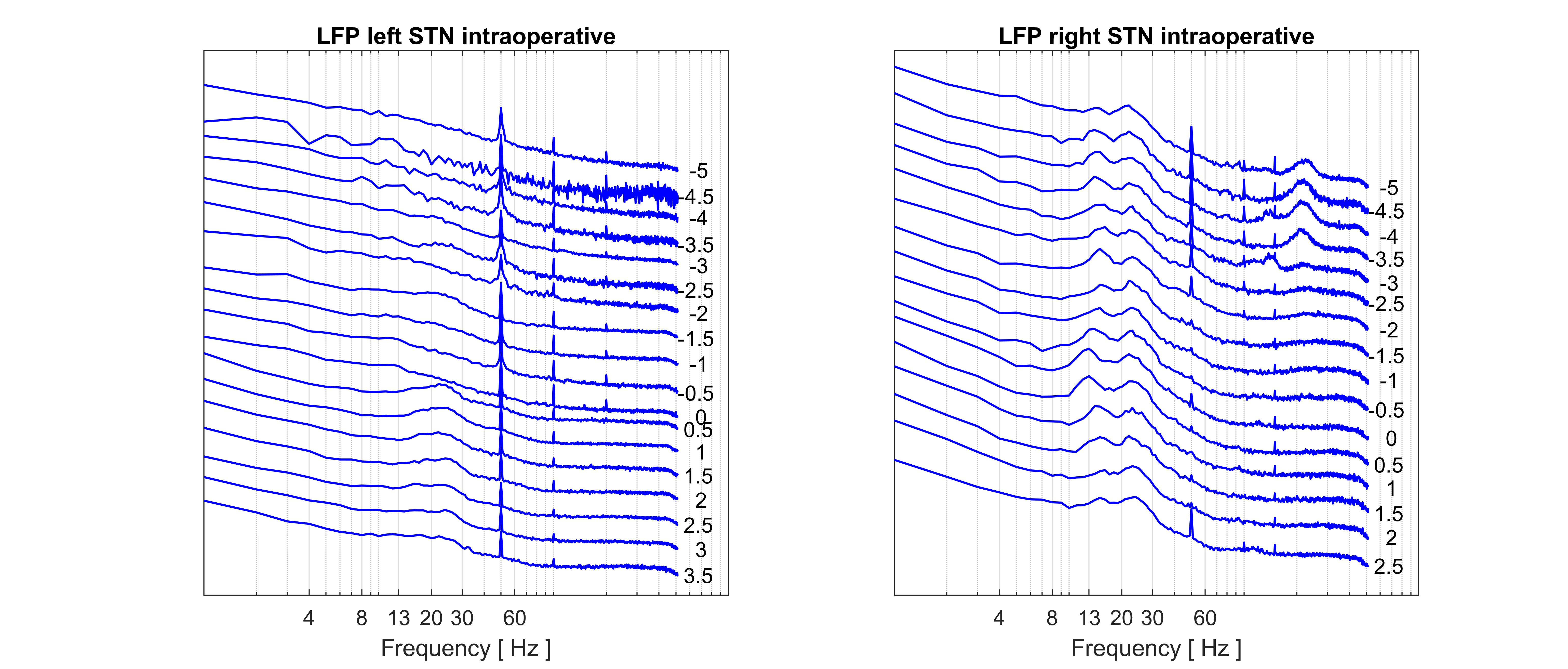

Supplement: Supplementary file 2 — High resolution image (TIFF 3876 kb) [file 701_2020_4493_MOESM1_ESM.tiff]
